# Use of Recombinant Mucin Glycoprotein to Assess the Interaction of the Gastric Pathogen *Helicobacter pylori* with the Secreted Human Mucin MUC5AC

**DOI:** 10.3390/bioengineering4020034

**Published:** 2017-04-15

**Authors:** Ciara Dunne, Anthony McDermott, Kumar Anjan, Aindrias Ryan, Colm Reid, Marguerite Clyne

**Affiliations:** 1 School of Medicine and Conway Institute of Biomolecular and Biomedical Science, University College Dublin, Belfield, Dublin 4, Ireland; ciaradunne51@gmail.com (C.D.); kumar.anjan@ucdconnect.ie (K.A.); aindrias.ryan@gmail.com (A.R.); marguerite.clyne@ucd.ie (M.C); 2 School of Veterinary Medicine, University College Dublin, Dublin 4, Ireland; anthony.mc-dermott@ucdconnect.ie (A.M.); colm.reid@ucd.ie (C.R.)

**Keywords:** MUC5AC, secreted mucin, *Helicobacter pylori*, glycosylation, protein secretion, gastric cells

## Abstract

There is intense interest in how bacteria interact with mucin glycoproteins in order to colonise mucosal surfaces. In this study, we have assessed the feasibility of using recombinant mucin glycoproteins to study the interaction of the gastric pathogen *Helicobacter pylori* with MUC5AC, a mucin which the organism exhibits a distinct tropism for. Stable clonal populations of cells expressing a construct encoding for a truncated version of MUC5AC containing N- and C-termini interspersed with two native tandem repeat sequences (N + 2TR + C) were generated. Binding of *H. pylori* to protein immunoprecipitated from cell lysates and supernatants was assessed. High molecular weight mucin could be detected in both cell lysates and supernatants of transfected cells. Recombinant protein formed high molecular weight oligomers, was both *N* and *O* glycosylated, underwent cleavage similar to native MUC5AC and was secreted from the cell. *H. pylori* bound better to secreted mucin than intracellular mucin suggesting that modifications on extracellular MUC5AC promoted binding. Lectin analysis demonstrated that secreted mucin was differentially glycosylated compared to intracellular mucin. *H. pylori* also bound to a recombinant C-terminus MUC5AC protein, but binding to this protein did not inhibit binding to the N + 2TR + C protein. This study demonstrates the feasibility of using recombinant mucins containing tandem repeat sequences to assess microbial mucin interactions.

## 1. Introduction

The tropism that bacteria display for distinct niches within host species is determined by the availability of specific receptors that the bacteria must interact with in order to colonise. The adhesins present on the bacteria that mediate binding to host receptors are often carbohydrate binding lectins which interact with specific glycosylated molecules present in the host. Such molecules include the heavily *O*-glycosylated mucin proteins present in mucus. Most bacterial interactions with mucins occur via the *O*-glycans that are attached to serine (Ser) and threonine (Thr) residues in variable number tandem repeated (VNTR) sequence motifs, the number of which varies between different mucins. Some bacteria bind directly to mucins [1,2], others use proteases or glycosidases to degrade the mucin so that they can penetrate the mucus layer and interact with the underlying epithelial cells [3]. Cross talk between bacteria and host cells can result in increased secretion of mucins [4,5] altered transcription of genes encoding for mucin [4,5] or alteration in glycosylation of mucins [5,6]. Studies have also shown that bacterial gene expression can be altered upon exposure to mucin [7,8].

Despite its obvious importance our knowledge of how bacteria interact with mucins has lagged behind that of the interaction with host cells. This is due in part to the fact that most cell culture systems do not produce a mucus layer and also the inherent difficulties in working with native mucins which are very large oligomeric glycoproteins. We have previously described a suite of MUC5AC mucin constructs and expressed them in airway mammalian cell lines [9]. These included constructs encoding for the N- and C-termini and for the N- and C-termini interspersed with two native tandem repeat (TR) sequences (N + 2TR + C). The N + 2TR + C construct resulted in a version of MUC5AC in which the size of the glycosylated VNTR domains and the molecular mass were both greatly reduced compared to that of native MUC5AC but the protein contained all of the functional domains required for the biosynthesis and secretion of a glycosylated mucin.

In this study, we aimed to generate a gastric cell line expressing the N + 2TR + C MUC5AC construct and use this model to examine the interaction of the gastric pathogen *Helicobacter pylori* with the recombinant form of the mucin. *H. pylori* colonises the hostile environment of the human stomach and displays a distinct tropism for the gastric mucin MUC5AC [10]. It is estimated that approximately 99% of *H. pylori* co-localise with either extracellular MUC5AC expressed in the mucus layer or the MUC5AC-producing epithelial cells highlighting the importance of the mucin in *H. pylori* colonization of the stomach [11,12]. The interaction of *H. pylori* with gastric mucin has been extensively studied. The organism binds MUC5AC via *O*-glycans expressed on the VNTR side chain of the mucin, including the Lewis b (Le^b^) carbohydrate structure [13], diacetyllactosediamine (LacdiNAc) [14] and sialylated structures such as sialyl-Lewis x and sialyl-Lewis a [15]. The outer membrane proteins BabA, SabA and LabA mediate binding to to Le^b^ [16], sialylated structures [15] and LacdiNAc [14] respectively. Thus *H. pylori* is a model organism for assessing the interaction of bacteria with mucins. We show that *H. pylori* binds to the recombinant form of MUC5AC produced by the cells, particularly the extracellular secreted mucin, which had an altered glycosylation profile compared to its intracellular form. This study demonstrates the feasibility of using recombinant mucin proteins to assess the direct interaction of bacteria with mucins and the mucin domains involved in mediating that interaction. 

## 2. Materials and Methods 

### 2.1. Cell Culture Conditions

The human gastric adenocarcinoma AGS cell line, was obtained from the American Type Culture Collection. Cells were maintained in Ham’s F12 medium (Lonza) supplemented with 10% (*v*/*v*) fetal bovine serum (FBS), 2 mM L-Glutamine and 1% (*v*/*v*) non-essential amino acids. Cells were routinely cultured in 75 cm^2^ tissue culture flasks (Greiner) at 37 °C and 5% CO_2_ in a humidified atmosphere.

### 2.2. Transfections

AGS cells were seeded at a density of 2 × 10^5^ cells/well and maintained in Ham’s F12 supplemented with 10% (*v*/*v*) FBS. The cells were grown for 30 h, at which time they had reached approximately 90% confluence. Cells were then transfected with 2 µg of DNA encoding for the MUC5AC N + 2TR + C construct using Lipofectamine^®^ LTX & Plus^TM^ Reagent (ThermoFisher Scientific, Paisley, UK) according to manufacturer’s instructions. After 18 h incubation, cell culture media was changed to Ham’s F12 + 10% FBS to allow for mucin expression. Clonal populations of AGS cells stably expressing the construct were isolated by selection with 40 μg/mL zeocin and hereafter are referred to as AGS MUC5AC cells. A clonal population of cells transfected with the C terminus construct was also generated as described above except that they were isolated by selection with 600 μg/mL G418. Control cells were transfected with an empty pcDNA3.1 plasmid and isolated as described above. All transfected cells were grown in the presence of selective antibiotic for plasmid maintenance.

### 2.3. Immunofluorescent Staining of Transfected AGS cells

Transfected AGS cells were grown on cover slips or transwell filters. Cells were washed with PBS to remove any non-adherent cells. The cells were fixed with 2% (*v*/*v*) formaldehyde in PBS and then permeabilised with 0.25% (*v*/*v*) Triton X-100 in PBS (PBST), each for 10 min at room temperature. For staining of non-permeabilised cells, treatment with Triton X-100 was omitted. Cells were blocked in PBS containing 1% (*wt*/*v*) bovine serum albumin (BSA; Sigma, Wicklow, Ireland) and 10% (*v*/*v*) goat serum (Sigma) for 1 h at room temperature. Cells were subsequently incubated with primary antibodies: anti-FLAG (1/215, Sigma), anti-His (1/50, Qiagen, Germany) or native MUC5AC 62M1 antibody, in blocking solution overnight at 4 °C. The 62M1 antibody detects epitopes in the D4, C and cysteine knot (CK) domains found in the C-terminus of the mucin [17], Following incubation with primary antibody, cells were probed with an anti-mouse secondary antibody conjugated to Alexa Fluor 488 (1/1000), (ThermoFisher Scientific, Paisley, UK). 4′,6-diamidino-2-phenylindole (DAPI, Sigma) was used to counterstain the nuclei of the cells (1/5000). Glass coverslips or transwell filters were mounted in fluorescent mounting medium (Agilent Technologies, Cork, Ireland) on glass microscope slides and sealed with nail varnish. Cells were visualized using an Olympus BX51 fluorescent microscope or a LSM710 confocal microscope (Zeiss, Welwyn Garden City, UK).

### 2.4. SDS-PAGE and Western Blotting

Transfected cells were rinsed in PBS to remove non-adherent cells and lysed in 100–250 μL CelLytic M (Sigma) at room temperature for 15 min with gentle agitation. Cells were scraped from the dish using a cell scraper and collected in Eppendorf tubes. The lysate was centrifuged at 17,000× *g* for 15 min to remove debris and the supernatant retained. Cell supernatants were screened for the presence of recombinant mucin. AGS MUC5AC cells were grown for up to 21 days in Ham’s F12 supplemented with 2% (*v*/*v*) FBS on 0.4 μm transwell filters, with media collected every two days. The cell culture media was concentrated using Amicon centrifugal filter units that had a 100 kDa molecular weight cut-off point. Reduced and non-reduced lysates and supernatants were analysed on either 6% (*v*/*v*) Bis-Tris acrylamide resolving gels or 3%–8% precast Tris-Acetate (TA) protein gels, 1.0 mm (Life Technologies). Proteins were then transferred for 18 h at 15 V and 4 °C to Immobilon-P Polyvinylidene Difluoride (PVDF) membrane (Millipore, Cork, Ireland). Membranes were probed with either Penta-His mononclonal antibody (1:2000, Qiagen), diluted in PBST containing 3% (*w*/*v*) BSA, or with anti-FLAG M2 monoclonal antibody (1:1000, Sigma) diluted in PBST containing 3% (*w*/*v*) skimmed milk powder. Finally, membranes were probed with a goat anti-mouse secondary antibody conjugated to horseradish peroxidase (HRP, Santa Cruz Biotechnology, Heidelberg, Germany), diluted 1/5000 in PBST and 3% (*w*/*v*) skimmed milk powder. Blots were developed using Enhanced Chemiluminescence (ECL, ThermoFisher Scientific, Paisley, UK).

### 2.5. Purification of Recombinant MUC5AC

Recombinant mucin produced and secreted by AGS cells was purified using M2 FLAG affinity agarose beads (Sigma) according to manufacturer’s instructions. Briefly, 40 μL of FLAG M2-affinity gel (Sigma) was incubated with 500–1000 μL of cell lysate or concentrated cell supernatant and incubated with rolling for 18 h at 4 °C. Recombinant N + 2TR + C or C-terminus protein bound to the beads was subsequently eluted by incubating the beads on a roller with 150 ng of 3× FLAG peptide for 45 min at 4 °C, which displaced the bound mucin by competitive inhibition. The resin was pelleted by centrifugation at 8000× *g* for 2 min and the supernatant recovered and stored at −80 °C. Purification using the His tag was performed using a PureProteome, Nickel Magnetic Bead System (Millipore), as per the manufacturer’s instructions.

### 2.6. Glycan Analysis

To examine the glycosylation of recombinant N + 2TR + C protein expressed by the AGS cells, protein purified by immunoprecipitation was run on a 3%–8% TA gel. Protein was transferred to a PVDF membrane as described above and probed with a panel of digoxigenin (DIG)-labelled lectins that recognize different sugar moieties from the DIG glycan differentiation kit (Roche, Table 1). To determine if the C-terminus protein was *N*-glycosylated it was treated with PNGase F (PROzyme) Enzyme Commission number (EC) 3.5.1.52, which removes *N*-glycans. Purified recombinant C-terminus protein was added to reaction buffer (100 mM sodium phosphate, pH 7.5) to yield a final concentration of 20 mM sodium phosphate in 45 μL total volume. Protein was denatured by adding 2.5 μL denaturation solution containing 2.0% (*wt*/*v*) sodium dodecyl sulfate (SDS) and 1M β-mercaptoethanol and incubated at 100 °C for 5 min. 2.5 μL of detergent solution containing 15% (*v*/*v*) Nonidet P-40 NP-40 detergent was added to the reaction solution. 1 μL of PNGase F enzyme (2.5 U/mL) was used per 100 μL of reaction mix. The reaction was incubated at 37 °C for 18 h. PNGase F treated and untreated C terminus protein was run on a 6% polyacrylamide sodium dodecyl sulfate (gel, transferred to PVDF membrane and probed with a His antibody. The C-terminus protein was also treated with Endo H (New England Biolabs, Hitchin, UK) which removes only high mannose and some hybrid types of N-linked carbohydrates. Protein was incubated with glycoprotein denaturing buffer (0.5% SDS and 0.04 M dithiothreitol (DTT)) and heated at 100 °C for 10 min. Reaction buffer (0.05 M sodium citrate, pH 5.5) and 1 µL of enzyme were added and the total volume adjusted to 20 µL. The reaction was incubated at 37 °C for 1 h. Endo H treated and untreated protein was separated on 6% polyacrylamide sodium dodecyl sulfate gel, transferred to PVDF and probed with *Galanthus nivalis* agglutinin (GNA) lectin.

### 2.7. Binding of H. pylori to Recombinant Mucin

A flow cytometric adherence assay was used to assess binding of *H. pylori* to recombinant mucin. *H. pylori* was cultured on Columbia blood agar base (Oxoid, Hampshire, UK) supplemented with 7% (*v*/*v*) horse defibrinated blood (Cruinn, Dublin, Ireland) and 0.8% (*v*/*v*) amphotericin B (250 μg/mL, HyClone, Buckinghamshire, UK). Inoculated plates were incubated in gas jars under microaerophilic conditions generated using Campygen gas packs (Oxoid) at 37 °C for 48 h. Once grown, bacteria were harvested from agar plates into Ham’s F12 tissue culture media and diluted to an O.D_600_ of 0.4 and 100 μL of bacteria was centrifuged. The bacterial pellet was then resuspended in 100 μL of Ham’s F12 containing 5%, 10% or 20% (*v*/*v*) of purified mucin. The bacteria were incubated in a 96-well dish in gas jars under microaerophilic conditions for 2 h at 37 °C. Following co-incubation with mucin, the bacteria were pelleted by centrifugation and the supernatant containing unbound mucin discarded. Bacteria were washed twice in PBS containing 0.05% (*v*/*v*) Tween 20 (washing buffer) to remove any unbound mucin, fixed in 2% formalin for 10 min, washed once and then blocked in PBS containing 1% BSA and 10% goat serum (Sigma) for 1 h at room temperature. To detect binding of the bacteria to the N + 2TR + C and the C-terminus recombinant proteins bacteria were probed with, a native MUC5AC antibody, 62M1 for 1 h at room temperature. The bacteria were washed three times and incubated with a goat anti-mouse secondary antibody conjugated to Alexa Fluor 488, diluted 1/2000 in PBS containing 1% BSA (*v*/*v*) for 1 h in the dark. Cells were washed three times and finally resuspended in 100 μL PBS. Cells were analysed on an Accuri C6 flow cytometer (BD BioSciences, Oxford, UK). Experiments were performed in compliance with the MIFlowCyt standard [18]. To assess if prior binding of the bacteria to the C-terminus protein could inhibit subsequent binding to the N + 2TR + C protein the bacteria were incubated with purified C-terminus protein prior to incubation with the N + 2TR + C protein. Binding of the bacteria to the N + 2TR + C protein was then assessed using flow cytometry as described above except that the 211M1 antibody, which detects the globular D/D2 domain in the N terminus of the protein [17], was used instead of the 62M1 antibody.

### 2.8. Statistical Analysis

All experiments were performed in triplicate on at least three separate occasions. The mean result of all experiments is given, with error bars indicating the standard deviation. 

## 3. Results

### 3.1. Generation of an AGS Clonal Cell Population Stably Expressing the N + 2TR + C MUC5AC Construct

**Figure 1:**
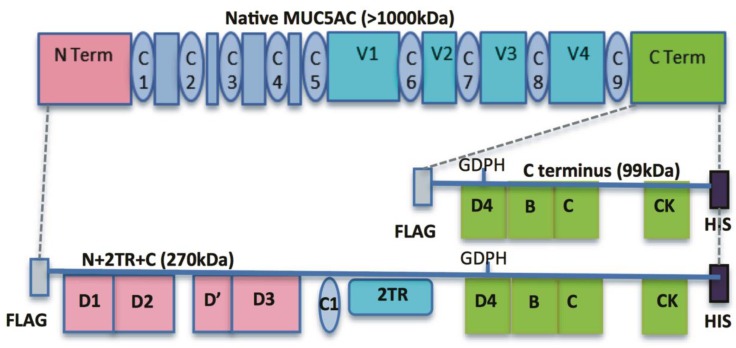
Diagrammatic representation of native MUC5AC and the C terminus and N + 2TR + C constructs used in this study (not to scale). The individual domains encoded for and location of the FLAG and His tags for each construct is shown. vWF-like domains (D1, D2, D’, D3, D4, B and C), Cys domains (C1–C9), cysteine knot (CK), variable number tandem repeat (VNTR) and tandem repeat (TR) regions are illustrated. The GDPH (Gly-Asp-Pro-His) domain in the D4 motif where post-translational cleavage of the C terminus occurs is indicated, as is the theoretical molecular mass of the translated apomucins (99 kDa for the C terminus protein and 270 kDa for the N + 2TR + C protein, however these values are peptide sequence only predicted molecular weights and the proteins are subject to post translational modifications which will alter the actual molecular weight.

A diagrammatic illustration of the N + 2TR + C and C terminus constructs is given in Figure 1. In the recombinant N + 2TR + C protein, two TR domains are found between the N- and C-terminus and these contain the amino acid sequence TTSTTSAP. A FLAG and His tag are incorporated into the constructs at the immediate N- and C-termini respectively, minimising the effect of the tags on mucin function while still allowing for easy purification and tracking of the recombinant protein. 

**Figure 2:**
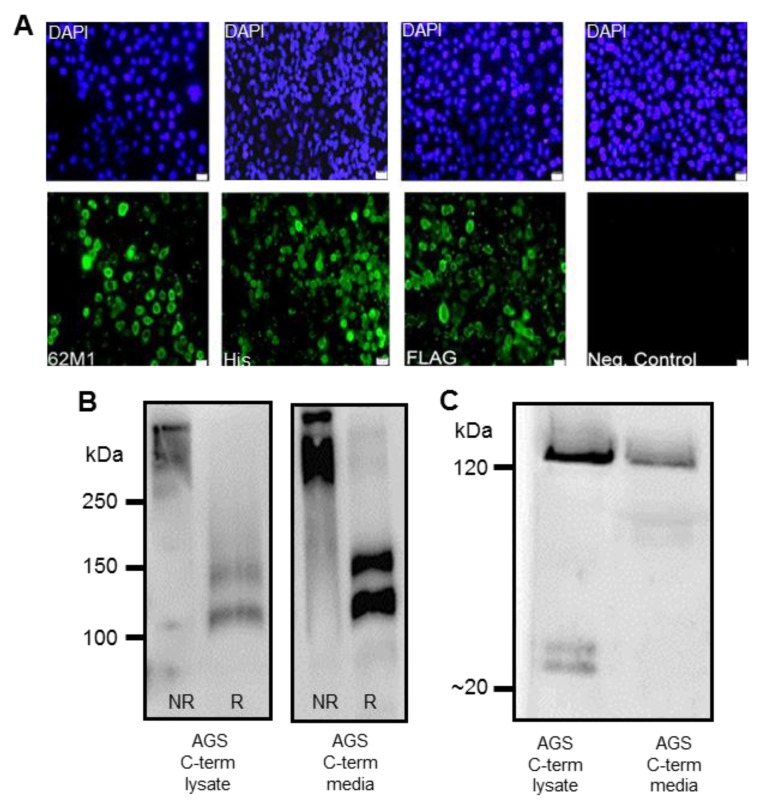
Expression of recombinant MUC5AC C terminus protein in the stably transfected human gastric adenocarcinoma AGS cell line. (**A**) Immunofluorescent staining of AGS cells transfected with the C terminus construct. Cell nuclei are stained with 4′,6-diamidino-2-phenylindole (DAPI) (blue) and expression of the C terminus protein (green) is detected with native MUC5AC antibody 62M1, FLAG antibody and His antibody. No staining was detected when cells were probed with secondary antibody in the absence of primary antibody (negative control); (**B**) The C terminus protein was detected in both cell lysate and supernatants (media) of AGS cells transfected with the C terminus construct. Protein samples were run on a gel containing 6% (*v*/*v*) Bis-Tris acrylamide under non reducing (NR) and reducing (R) conditions, transferred to a Polyvinylidene Difluoride (PVDF) membrane and probed with a His antibody; (**C**) Western blot of concentrated and His immuno-precipitated MUC5AC C-terminus from cell lysates and from culture supernatant (media) under reducing conditions probed with FLAG antibody. A ~120 kDa band corresponds to uncleaved C-terminus protein. Lower >20 kDa bands are cleavage products extending from the N terminus of the MUC5AC C-terminus construct to the GDPH cleavage site.

**Figure 3:**
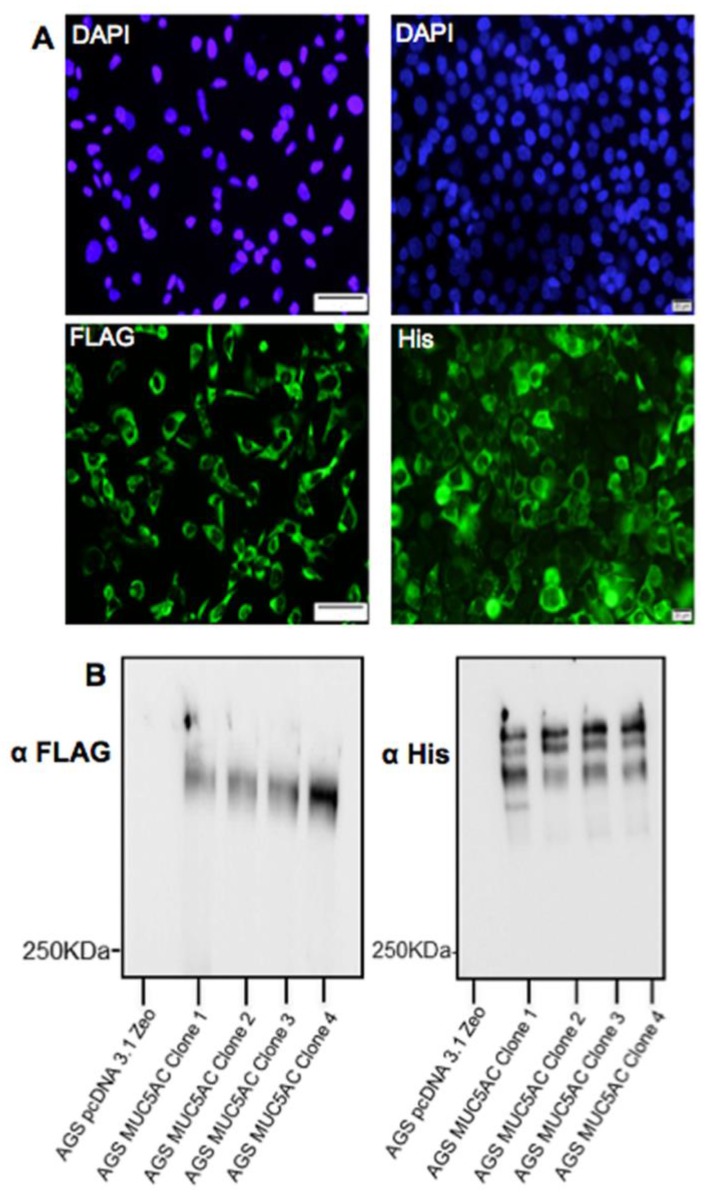
Expression of recombinant MUC5AC N + 2TR + C protein in stably transfected AGS cells. (**A**) Representative images of immunofluorescent staining of AGS N + 2TR + C cells expressing the N + 2TR + C construct. Cells were probed with either FLAG or His antibody and a goat anti mouse secondary antibody conjugated to Alexa Fluor 488 to detect mucin expression (green). Cells were counterstained with 4′,6-diamidino-2-phenylindole (DAPI) (blue) to detect cell nuclei; (**B**) Western Immunoblotting of cell lysates of four clonal populations of AGS N + 2TR + C cells. Non-reduced samples were separated on 6% polyacrylamide sodium dodecyl sulfate gels, transferred to PVDF and probed with either FLAG or His antibody. There was no protein detected in a lysate of cells transfected with an empty pcDNA3.1 plasmid.

Clonal isolates of stably transfected cells were selected and expression of the recombinant protein was confirmed using immunofluorescent staining and western immunoblotting. Western immunoblotting confirmed that the recombinant products expressed in each of the clonal populations reacted with both the FLAG and His antibodies, indicating that the full-length constructs were being expressed (Figure 2 and Figure 3). Detection of bands with a higher molecular weight than the predicted molecular weights of ~99 and ~270 kDa for the C-terminus and N + 2TR + C proteins respectively suggest that *N*- or *O*-glycosylation of the immature apomucin may be occurring, generating products of larger size. Stable clonal populations of cells transfected with the C-terminus construct reacted with the 62M1 antibody, which detects an epitope present on the C-terminus of native MUC5AC, and with FLAG and His antibodies (Figure 2A). Staining of a large proportion of the cells with the FLAG and His antibodies respectively also suggests that full length protein is likely to be expressed in these cells. It has previously been shown that the C terminus of MUC5AC forms dimers through its CK domain and undergoes auto-catalytic cleavage in a Gly-Asp-Pro-His (GDPH) sequence [19]. Western blotting and probing with a His antibody of purified C-terminus protein under reducing and non-reducing conditions from both cell lysates and cell supernatants was used to determine if these events also occurred in the AGS cells. Under non-reducing conditions a product of approximately 250 kDa was detected indicating that dimerization was occurring (Figure 2B). Bands of approximately 120 kDa and 100 kDa corresponding to the C-terminus and a C terminal cleavage product were detected under reducing conditions (Figure 2B). There are 23 potential N-glycosylation sites on the C-terminus protein and the increase in molecular weight from that predicted is probably due to the presence of N-glycosylation. His immuno-precipitated MUC5AC C-terminus from cell lysates and from culture supernatant (media) probed with FLAG antibody revealed uncleaved C-terminus protein and low molecular weight (~20–25 kDa) cleavage products extending from the N terminus of the MUC5AC C-terminus construct to the GDPH site (Figure 2C). 

Four clonal populations of AGS MUC5AC cells expressing the N + 2TR + C construct were isolated. Recombinant protein expression could be detected by immunofluorescent staining (Figure 3A) and western immunoblotting (Figure 3B). Probing of western blots under non-reducing conditions showed some differences in the molecular weight of the proteins detected with the FLAG antibody in the different clones. These variations are likely due to differences in glycosylation density and length. The presence of multiple bands detected with the His antibody suggested that the mucin was undergoing post-translational modifications. Clone 1 was selected and used for all subsequent experiments. 

### 3.2. Secretion and Oligomerisation of Recombinant N + 2TR + C Mucin

**Figure 4:**
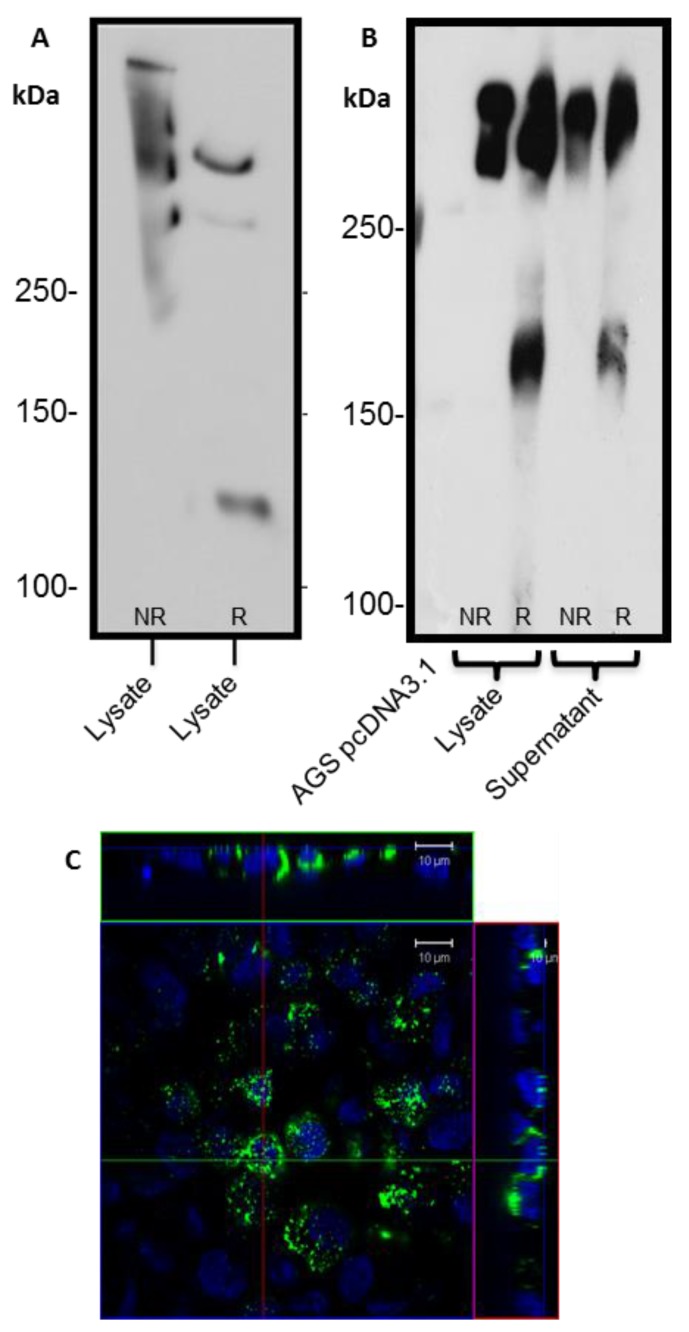
Oligomerisation and secretion of N + 2TR + C MUC5AC protein by AGS cells. (**A**,**B**) Recombinant protein was purified from cell lysates and cell supernatants by immunoprecipitation. Reduced (R) and non-reduced (NR) forms of purified mucin were separated on (**A**) 6% SDS PAGE gels or (**B**) 3–8% TA gels, transferred to PVDF and probed with an anti-His antibody; (**C**) 21 day old non-permeabilised AGS MUC5AC cells were probed with anti-His antibody and a goat anti-mouse secondary antibody conjugated to AlexaFluor 488 to reveal mucin (green). Cell nuclei were counterstained with DAPI (blue). Orthographic projection of AGS MUC5AC cells is given, demonstrating the presence of mucin overlying the cell nucleus and between the cells.

Analysis of N + 2TR + C protein expressed by AGS cells under reducing and non-reducing conditions revealed that the protein was undergoing oligomerisation (Figure 4A). The reduced form of recombinant MUC5AC migrated further than the non-reduced form, indicating that oligomerisation of the N + 2TR + C MUC5AC construct occurred in the AGS cells. A band of approximately 120 KDa was seen when reduced lysates were probed with an anti-His antibody (Figure 4A), which is likely to represent post-translational cleavage of the mucin. Protein could also be detected in the cell supernatant demonstrating that mucin was being secreted from the cells (Figure 4B). Interestingly when we ran our samples on Tris acetate gels under reducing conditions (Figure 4B) we detected a band of ~160 kDa band in addition to higher molecular weight material. We could also detect a 120 kDa band in this material upon prolonged exposure (Figure S1). Confocal microscopy of non-permeabilised cells additionally illustrated that small amounts of extracellular mucin could be detected adherent to the cell surface (Figure 4C).

### 3.3. Glycan Analysis of Intracellular and Secreted Recombinant Mucin

**Figure 5:**
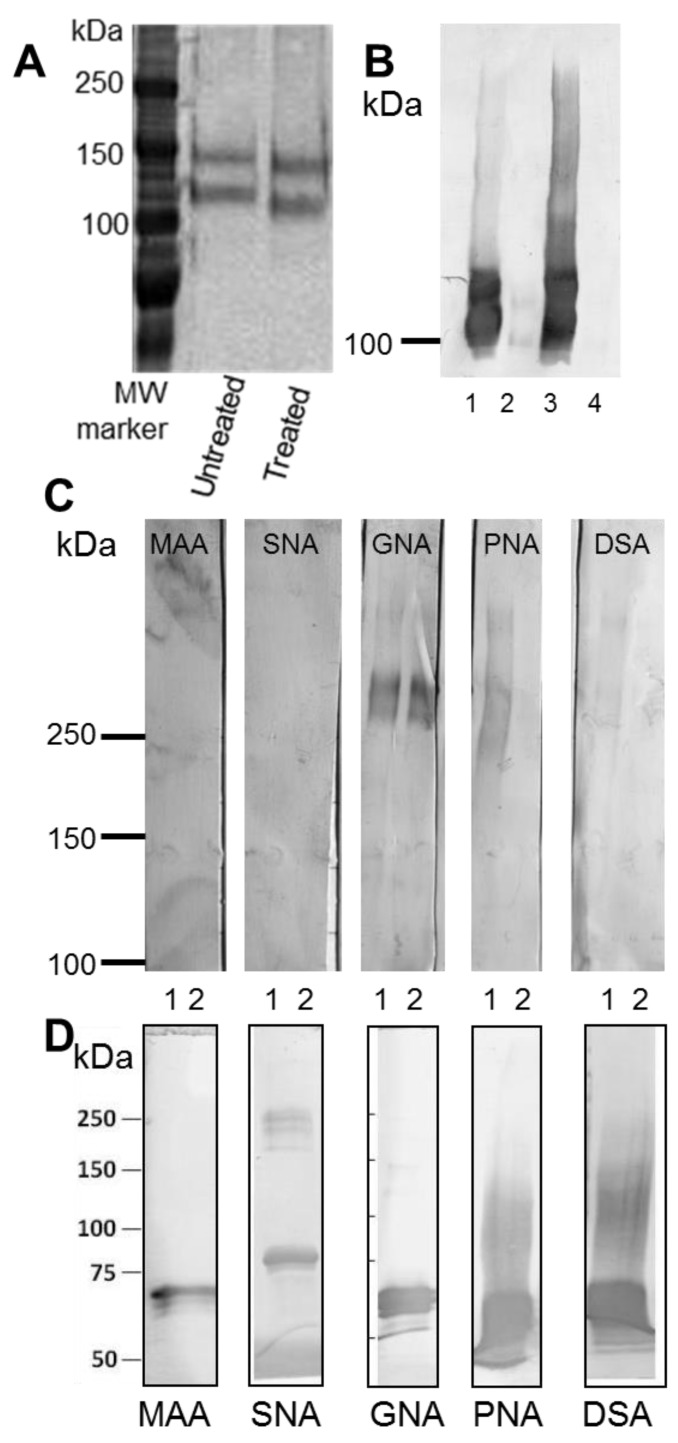
Glycan analysis of intracellular and secreted recombinant C terminus protein and N + 2TR + C MUC5AC protein. (**A**) Treatment of the C terminus protein with PNGase F resulted in a reduction in molecular weight of the protein detected with a His antibody indicating that this protein is *N*-glycosylated; (**B**) Recombinant C terminus purified from cell lysates and cell culture media, treated or untreated with Endo H was separated on 6% acrylamide gel, transferred to PVDF and probed with *Galanthus nivalis* agglutinin (GNA) lectin. Lane 1: AGS C-term lysate untreated. Lane 2: AGS C-term lysate treated with endoglycosidase H. Lane 3: AGS C-term media untreated. Lane 4: AGS C-term media treated; (**C**) N + 2TR + C recombinant mucin was run on 3%–8% TA gels and transferred to PVDF. Membranes were incubated with five digoxigenin-labelled lectins *Maackia amurensis* (MAA), *Sambucus nigra* agglutinin (SNA), GNA, Peanut agglutinin (PNA) and *Datura stramonium* agglutinin (DSA). Lane 1: N + 2TR + C mucin purified from cell supernatant. Lane 2: N + 2TR + C mucin purified from cell lysate; (**D**) Positive control blots for each lectin are shown. Fetuin was probed with MAA, Transferrin was probed with SNA, Carboxypeptidase Y was probed with GNA and Asialofetuin was probed with PNA and DSA.

There are 23 potential glycosylation sites on the C terminus protein. Treatment of the C terminus protein with glycosidases PNGase F (Figure 5A) and endoglycosidase H (Figure 5B) resulted in a reduction in molecular weight (Figure 5A) and loss of reactivity with GNA lectin (Figure 5B) indicating that this protein was *N*-glycosylated. The N + 2TR + C protein contains 2 TRs and so there is the potential for *O*-glycosylation to also occur. Glycosylation of the N + 2TR + C mucin purified from both cellular lysates of transfected cells and cell culture supernatant was assessed for reactivity with a panel of lectins. Reactivity was detected with lectins GNA Peanut agglutinin, (PNA) and *Datura stramonium* agglutinin (DSA) (Figure 5C). GNA which detects mannose-mannose linkages reacted with N + 2TR + C protein purified from cell lysate and cell supernatant, indicating that both the immature intracellular and secreted forms of recombinant N + 2TR + C protein were *N*-glycosylated. The lectins PNA and DSA (which react with *N*-acetylgalactosamine (GalNAc) residues in *O*-glycans and galactose (1,4)-GalNAc linkages in *N*- and *O*-linked glycans as well as *N*-acetylglucosamine residues expressed in *O*-glycans respectively) only bound to secreted forms of the protein, suggesting that the extracellular mucin was differentially glycosylated compared to mucin in cell lysates. The reactivity of the secreted but not the intracellular N + 2TR + C protein with lectins PNA and DSA indicated that the 2TR domain was functional in the AGS cells (Figure 5). Although it has been reported that sialylation is increased in gastric tumour tissue compared to normal gastric tissue [20] no signal was detected when mucin purified from either cell lysates or supernatants was probed with *Maackia amurensis* agglutinin (MAA) or *Sambucus nigra* agglutinin-I (SNA-I) suggesting that sialylation of the N + 2TR + C protein was not occurring. 

### 3.4. Interaction of Helicobacter pylori with Recombinant MUC5AC

**Figure 6:**
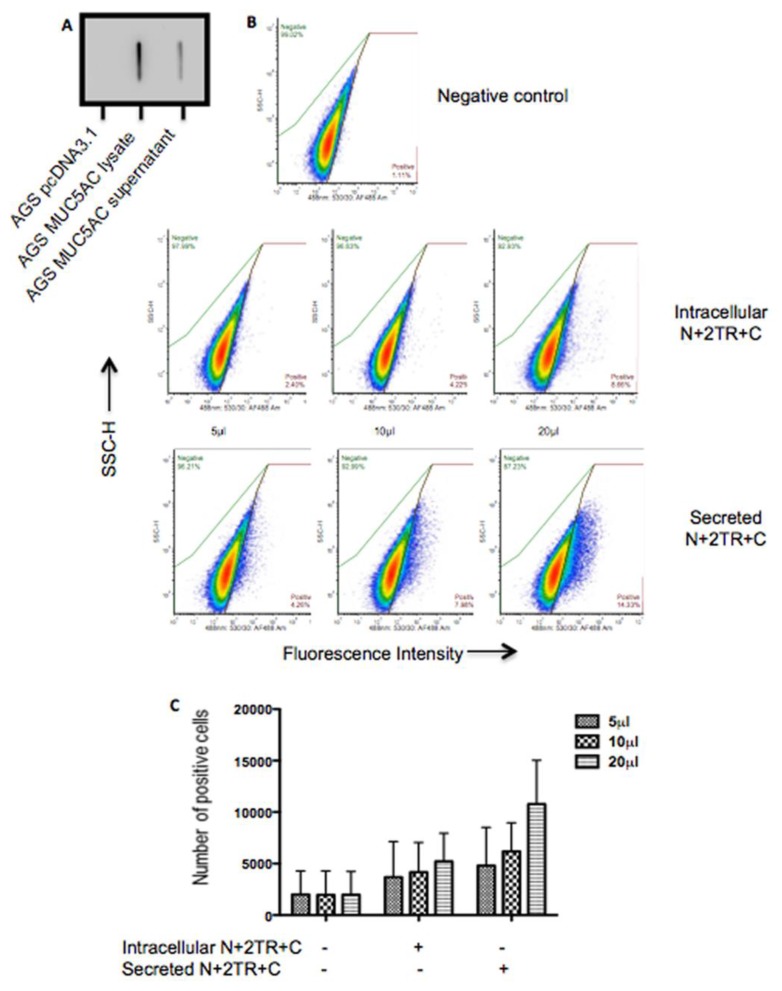
Binding of *H. pylori* to intracellular and secreted forms of recombinant N + 2TR + C MUC5AC produced by AGS cells. (**A**) Immunoblotting of N + 2TR + C mucin purified from cell lysate and cell supernatant, demonstrating that a lower concentration of secreted protein was immunoprecipitated; (**B**) Scatter blots illustrating a shift in fluorescence upon incubation with mucin. As a negative control bacteria were incubated with immunoprecipitate from AGS cells transfected with pcDNA3.1 plasmid only. The height and area of bacterial cells were plotted against each other and gated to ensure that only single cells were analysed. The level of fluorescence of single cells upon incubation with immunoprecipitate from AGS pcDNA3.1 lysate (negative control), AGS N + 2TR + C lysate (intracellular) and AGS N + 2TR + C cell supernatant (secreted) was measured; (**C**) Graphical representation of the number of positive fluorescent cells (*y*-axis) upon incubation with purified protein from different sources (*x*-axis). The average of three biological replicates is shown, with error bars representing the mean ± standard deviation.

A flow cytometric adherence assay was used to assess the interaction of *H. pylori* with recombinant MUC5AC proteins. Bacteria were incubated with protein that had been purified by FLAG-based immunoprecipitation. This method yields pure but low concentrations of protein that are below the limit of detection of standard protein assays such as Bradford Assay. For this reason, it was not possible to measure the exact concentration of protein used. Therefore equal volumes of purified intracellular and extracellular protein were slot blotted onto a membrane and probed with a His antibody. A less intense signal was obtained with secreted mucin than with mucin purified from cell lysates indicating that the concentration of stock purified extracellular mucin was less than that of stock intracellular mucin (Figure 6A). *H. pylori* was incubated with increasing amounts of purified intracellular and extracellular mucin for two hours at 37 °C, and bacterial cells were probed with a MUC5AC-specific antibody. *H. pylori* bound to both intracellular and secreted forms of N + 2TR + C protein produced by the AGS cells. However despite the fact that a lower concentration of secreted protein was used compared to the intracellular form, enhanced binding to the extracellular form was detected in every instance (Figure 6B,C and Table 2). These results suggest that modifications on the secreted form of MUC5AC produced by AGS cells promoted *H. pylori* binding. 

### 3.5. Binding of H. pylori to the C-Terminus of MUC5AC

**Figure 7:**
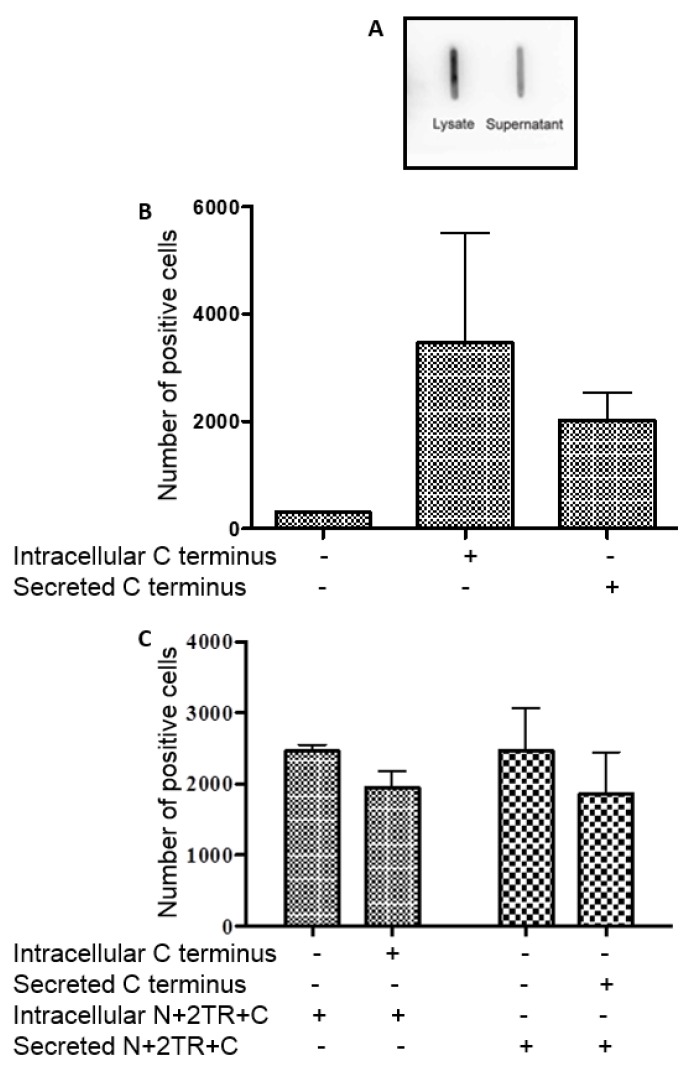
Interaction of *H. pylori* with the C-terminus of MUC5AC. (**A**) Slot Blot showing the presence of recombinant C-terminus protein in immunoprecipitations from cell lysate and from cell culture supernatant. Samples were blotted onto PVDF membrane and probed with an anti-His antibody to detect the presence of recombinant protein; (**B**) Binding of *H. pylori* P12 to intracellular and secreted forms of MUC5AC C-terminus; (**C**) Binding of *H. pylori* to intracellular and secreted forms of N + 2TR + C protein in the presence of intracellular and secreted forms of C terminus protein.

To further explore how *H. pylori* binds recombinant N + 2TR + C protein, recombinant C terminus protein was purified from both lysates and supernatants of AGS cells stably transfected with the C terminus construct. Probing of purified protein slot blotted onto a membrane with a His antibody demonstrated that the concentration of intracellular mucin used was higher than the concentration of secreted protein (Figure 7A). Flow cytometry was used to detect binding of *H. pylori* with equal volumes of recombinant C-terminus intracellular and secreted protein. *H. pylori* bound to both the intracellular and secreted form of the C-terminus protein (Figure 7B). The effect of C terminus protein on binding of *H. pylori* to recombinant N + 2TR + C protein was then assessed. Pre-incubation of *H. pylori* with either the intracellular or secreted form of C terminus protein prior to incubation of the bacteria with N + 2TR + C protein did not result in a reduction in binding to the N + 2TR + C protein, indicating that with the N + 2TR + C protein that in addition to binding to the C terminus of the protein binding can also occur at other sites. Alternatively lack of effect of C terminus protein on binding of *H. pylori* to N + 2TR + C protein might reflect a higher affinity of the organism for the N + 2TR + C protein.

## 4. Discussion

Molecular manipulation of mucins has always been difficult due to their large size and the repetitive nature of the sequences that encode the *O*-glycosylation domains. We have previously reported expression and characterisation of the MUC5AC N + 2TR + C protein in respiratory cell lines [9]. Prior to this only individual mucin domains had been expressed in mammalian cells. In this study we report expression of the N + 2TR + C protein in gastric AGS cells, further underlining the feasibility of producing recombinant secreted mucins in mammalian cell lines. In addition we also demonstrate the interaction of *H. pylori*, a gastric pathogen with a particular tropism for MUC5AC, with the recombinant protein. Our results suggest that production of recombinant mucins containing N and C termini and tandem repeat regions in different cells should provide a novel platform that allows for detailed analysis of mucin biosynthesis, secretion and function in different settings. In this study we specifically demonstrate the use of the purified recombinant proteins to assess binding of *H. pylori* and the role of specific mucin domains in mediating that binding. 

In the human stomach the secreted mucins MUC5AC and MUC6 are both expressed. Purification of mucin from native material is therefore very likely to contain a mixture of different types of mucin. An advantage of expressing recombinant mucin in cells is that homogenous material can be easily isolated. However the N + 2TR + C protein used in this study differs from native MUC5AC in terms of the number of tandem repeat domains expressed by the protein (2 vs. > 50–100). The tandem repeat regions of mucins are unique in terms of size, sequence and number of repeats. The VNTR region of *muc* genes that encodes for the repeat region is highly polymorphic, which leads to differences between individuals in terms of the number of repeats contained within a mucin [21]. The VNTR region is the site of *O*-glycosylation, where carbohydrate chains are attached to threonine or serine residues within the polypeptide chain in an *O*-linked manner. Mucin glycosylation is a dynamic process and is also tissue specific. A recent study looking at glycosylation of gastric tissue from different individuals demonstrated that *O*-glycans were dominated by neutral and fucosylated structures. The diversity of *O*-glycans reflected the presence of blood type ABH epitopes, and i/I branches as well as terminal α1,4-GlcNAc like and GalNAcβ1-4GlcNAc (LacdiNAc)like structures [20]. In line with our previous study with the N + 2TR + C construct [9] we found that the reduced number of TR domains did not affect the processing of the mucin in the AGS cells, with complete trafficking as well as *N*-glycosylation and possibly *O*-glycosylation of the recombinant protein seen in the stably transfected cells. While *O*-glycosylation of recombinant protein may be affectedby the presence of only two TR domains, there are eight Thr and four Ser residues incorporated into the TR region, providing twelve potential *O*-glycosylation sites. Although the AGS cells are a gastric adenocarcinoma cell line we did not detect any reactivity of the N + 2TR + C protein with the lectins MAA or SNA-1. This was surprising as it has recently been shown that gastric tumour tissue demonstrates increased sialylation compared to gastric tissue from healthy controls [20]. However the same study also reported that there was an enormous diversity in glycosylation between individuals thus making it difficult to identify gastric cancer specific structures [20]. 

Detection of the C-terminus and the N + 2TR + C protein using both FLAG and His antibodies suggested that full-length proteins were expressed in stably transfected cells. Difficulties in obtaining stable populations that secrete recombinant mucins have been reported [22]. However western immunoblotting clearly showed that the AGS cells were capable of secreting both C terminus protein and N + 2TR + C protein. The extracellular form of mucin is the more mature form, having undergone complete trafficking through the cell before being secreted and can often differ from intracellular mucin, which has not been fully processed [23]. For a complete review on the sequence of events in the biosynthesis of mucins, mucin core structures, backbone repeat glycans and peripheral sequences found on glycans see reference [24]. The predicted size of the N + 2TR + C protein, based on the amino acid sequence, was 270 kDa. Detection of protein with a higher molecular weight suggests that post-translational modifications were occurring. This is supported by (1) protein oligomerisation and (2) the extracellular form of the protein when compared to the immature intracellular protein, displayed N and possibly *O*-glycosylation. In addition to high molecular weight products, a band of approximately 120 kDa was detected in lysates probed run on SDS polyacrylamide gels with the anti-His antibody. This pattern is indicativeof cleavage of the mucin in the GDPH sequence located in the C-terminus reported previously [19]. Thus characterisation of the N + 2TR + C protein in terms of cleavage, oligomerisation, secretion and glycosylation illustrated that the AGS cells were processing the protein in a similar manner to that of native MUC5AC. In addition to the 120 kDa cleavage product we also detected a band at ~160 kDa when purified N + 2TR + C protein from both cell lysate and cell supernatant was run on Tris acetate gels under reducing conditions (Figure 4). We do not know what this band is but it may be another MUC5AC cleavage product as a proteomic analysis of MUC5AC secreted by the airway epithelial cell line, Calu-3 has identified up to 16 potential cleavage sites [25]. Lack of detection of this band when 6% Bis-Tris gels are used may be due to the amount of protein loaded on the gel and/or better separation of large molecular weight proteins on 3%–8% NuPAGE Tris acetate gels. 

*H. pylori* binding of native MUC5AC is mediated by glycans [13,14,26]. Increased numbers of bacteria bound to the secreted N + 2TR + C protein than bound to the intracellular form. The higher level of binding to the secreted form of MUC5AC may be due to the expression of *O*-glycans on the protein. Thus *H. pylori* likely interacts with the recombinant MUC5AC produced and secreted by AGS cells via carbohydrate structures expressed on the mucin.

*H. pylori* also interacted with the recombinant C-terminus protein. This section of the mucin undergoes mainly *N*-glycosylation, with few if any *O*-glycosylation sites expressed in this part of the MUC5AC protein [25]. We demonstrated using PNGaseF treatment of this C-terminus protein that the recombinant mucin was *N*-glycosylated. While most studies have focused on and identified *O*-glycan receptors of *H. pylori*, it is possible that the pathogen could bind to *N*-glycans too and so these, as well as the protein back-bone, may potentially serve as receptors for the bacteria. However the C terminus protein was unable to inhibit binding of *H. pylori* to the N + 2TR + C protein indicating that the bacteria exhibit a greater affinity for this protein than they do for the C terminus alone. 

*H. pylori* also interacted with the recombinant C-terminus protein. This section of the mucin undergoes mainly *N*-glycosylation, with few if any *O*-glycosylation sites expressed in this part of the MUC5AC protein [25]. We demonstrated using PNGaseF treatment of this C-terminus protein that the recombinant mucin was *N*-glycosylated. While most studies have focused on and identified *O*-glycan receptors of *H. pylori*, it is possible that the pathogen could bind to *N*-glycans too and so these, as well as the protein back-bone, may potentially serve as receptors for the bacteria. However the C terminus protein was unable to inhibit binding of *H. pylori* to the N + 2TR + C protein indicating that the bacteria exhibit a greater affinity for this protein than they do for the C terminus alone. 

We have generated a gastric cell line that stably expresses recombinant MUC5AC and shown that the cells process the mucin in a similar manner to that of native MUC5AC, including post-translational cleavage of the C-terminus, oligomerisation, glycosylation and secretion. However there are several limitations to our system. A bead-based immunoprecipitation method was used to purify recombinant protein, and, although efficient, this method yielded only small amounts of recombinant product that, despite concentration, was unfortunately too low to quantify. In order to produce larger amounts of protein it will be necessary to look at alternative culture systems such as hollow fiber systems where the cells can be grown for long periods of time and the culture media easily collected. This would allow for a more accurate analysis of glycosylation using mass spectrometry. Detailed analysis of glycosylation would enable identification of potential binding sites for the bacteria and for identification of glycans that could be used in competitive inhibition experiments. Larger amounts of material would also allow for alternative methods such as enzyme-linked immunosorbent assay (ELISA)-like binding assays to be used if flow cytometry was not available. Glycosylation in cell lines is normally less complex compared to glycosylation that occurs in vivo. However manipulation of the cells by transfection with specific glycosyltransferase constructs should allow for production of cells that produce biologically relevant material. Similar to native MUC5AC, *H. pylori* organisms bound to the recombinant mucin protein, displaying particular affinity for the mature glycosylated form of the N + 2TR + C protein indicating that the material produced by the AGS cells contained glycans that the bacteria could interact with.

The interaction of bacteria with mucin is currently an area of keen investigation. This is due in part to the large number of recent studies which highlight the important role of the gastrointestinal microbiome in maintaining health and wellbeing and the association of dysbiosis with disease [27]. The gut microbiome colonises the mucus layers overlying the intestinal epithelial cells [28]. Knowledge of how bacteria interact with and colonise mucus should lead to development of strategies to enhance colonisation with probiotic or beneficial bacteria that promote health and prevent infection with pathogens.

## 5. Conclusions

Several investigators have used mucin secreting cell lines such as the various subclones of the HT-29 cell line to assess not just direct bacteria host binding but also the effect of mucin expression on host-microbial cross talk [29,30,31]. The development of constructs which allow for expression of truncated mucins containing TR regions should allow for the development of cell lines that secrete biologically relevant mucins and more closely mimic the in vivo environment. The ease with which these proteins can be purified and also mutated will facilitate more detailed studies on mucin biology. This study demonstrates the feasibility of using recombinant mucins containing tandem repeat sequences to assess microbial mucin interactions however, the stably transfected cells described here could potentially be used to explore the role of MUC5AC in a number of other aspects of infection including the effect of organisms similar to *H. pylori* on mucin processing and glycosylation.

## Figures and Tables

**Table 1:** Lectins used in digoxigenin (DIG)-glycan analysis and respective epitopes.

Lectin	Name	Epitope
GNA	*Galanthus nivalis* agglutinin	Terminal mannose, α(1-3) or α(1-2) to mannos
SNA-I	*Sambucus nigra* agglutinin	Sialic acid linked α(2-6) to galactose
MAA	*Maackia amurensis* agglutinin	Sialic acid linked α(2-3) to galactose
PNA	Peanut agglutinin	Galactose linked β(1-3) to *N*-acetylglucosamine
DSA	*Datura stramonium* agglutinin	Gal-β(1-4)GlcNAc (*N*-glycans) and GlcNAc (*O*-glycans)

**Table 2:** Median fluorescent intensity of the negative and positive bacterial populations from Figure 6B

Source of Mucin	All	Positive		Negative
	Count	% of this plot	Median Fluorescent Intensity	Median Fluorescent Intensity
**AGS pcDNA3.1 Zeo lysate**	103,048	1.11%	N/A	442
**5 µl AGS N + 2TR + C lysate**	102,490	2.4%	6,039	403
**10 µl AGS N + 2TR + C lysate**	102,210	4.22%	6,701	548
**20 µl AGS N + 2TR + C lysate**	102,842	8.66%	5,941	594
**5 µl AGS N + 2TR + C supernate**	102,630	4.20%	6,301	469
**10 µl AGS N + 2TR + C supernate**	102,404	7.98%	6,753	542
**20 µl AGS N + 2TR + C supernate**	102,344	14.33%	7,102	588

## Supplementary Materials

The following are available online at http://www.mdpi.com/2306-5354/4/2/34/s1. Figure S1: oligomerisation and secretion of N + 2TR + C MUC5AC protein by AGS cells. Recombinant protein was purified from cell lysates and cell supernatants by immunoprecipitation. Reduced (R) and non-reduced (NR) forms of purified mucin were separated on 3%–8% TA gels, transferred to PVDF and probed with an anti-His antibody. Prolonged exposure of this blot was necessary to reveal detection of lower molecular weight cleavage products (100–120 kDa) that was readily detected when the samples were run on a 6% SDS PAGE gel (Figure 4A).
