# Supplementary material for: Use of Recombinant Mucin Glycoprotein to Assess the Interaction of the Gastric Pathogen Helicobacter pylori with the Secreted Human Mucin MUC5AC
Source: Bioengineering (Basel). 2017 Apr 15;4(2):34. doi: 10.3390/bioengineering4020034 (PMC5590460; doi:10.3390/bioengineering4020034)
Supplement: Supplementary file 1 [file bioengineering-04-00034-s001.pdf]

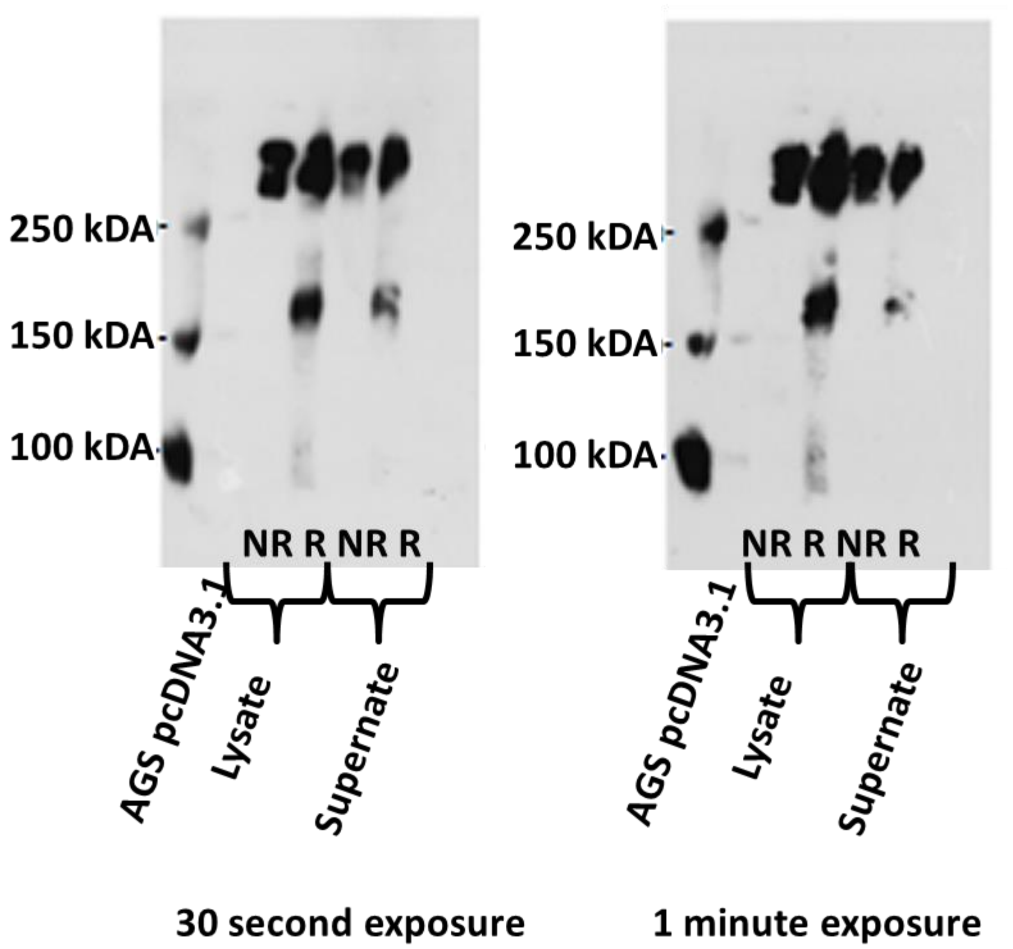

Figure S1. Oligomerisation and secretion of N+2TR+C MUC5AC protein by AGS cells. Recombinant protein was purified from cell lysates and cell supernatants by immunoprecipitation. Reduced (R) and non-reduced (NR) forms of purified mucin were separated on 3-8% TA gels, transferred to PVDF and probed with an anti-His antibody. Prolonged exposure of this blot was necessary to reveal detection of lower molecular weight cleavage products (100-120 kDa) that was readily detected when the samples were run on a 6% SDS PAGE gel (Figure 4A).
